# Effectiveness of mechanical treatment with customized insole and minimalist flexible footwear for women with calcaneal spur: randomized controlled trial

**DOI:** 10.1186/s12891-022-05729-4

**Published:** 2022-08-13

**Authors:** Ana Paula Ribeiro, Brenda Luciano de Souza, Silvia Maria Amado João

**Affiliations:** 1 Physical Therapy Department, Post-Graduate Department, School of Medicine, University of São Paulo, R: Cipotânea, 51, Campus Universitário, São Paulo/SP, Brazil; 2 School of Medicine, Post-Graduate in Health Science Department, Biomechanics and Musculoskeletal Rehabilitation Laboratory, University Santo Amaro, São Paulo, Brazil

**Keywords:** Foot, Pain, Insoles, Footwear, Gait, Training, Calcaneus spur

## Abstract

**Backround:**

Calcaneal spurs are described as bony outgrowths arising on medial calcaneal, where inappropriate footwear can promote disease progression.

**Objective:**

Investigate the effectiveness of mechanical treatment with customized insole and minimalist flexible footwear during gait training program in women with calcaneal spur.

**Methods:**

Design: A single-blinded, randomized and controlled trial. Setting: Biomechanics laboratory. Participants: Forty-three women, 29 with calcaneal spur and 14 control.

**Intervention:**

Gait training program with use of the minimalist flexible footwear (MFG *n* = 15, age: 48.9 ± 9.4, height: 1.61 ± 0.1, BMI: 32.1 ± 7.0) and customized insole on footwear (COIG *n* = 14, age: 50.3 ± 5.8, height: 1.62 ± 0.1, BMI: 32.2 ± 4.3) and control (CG *n* = 14, age: 47.8 ± 8.6, height: 1.63 ± 0.1, BMI: 27.5 ± 4.5), followed of the evaluations: baseline (T0) and after three (T3) and six (T6) months. Duration of the intervention was of the six months consecutive for at least 42 h per week (six hours a day, seven days a week). Outcome primary were calcaneus pain (visual analogue scale), Foot Function Index (FFI), Foot Health Status Questionnaire (FHSQ-Br) and 6-min walk test (6MWT). Secondary was plantar pressure distribution by a pressure platform system during gait and static index foot posture (FPI). Statistical analysis: analysis of variance for repeated measure and between groups were used to detect treatment-time interactions (α = 5%). Effect size with D Cohen’s also was used between T0 and after six (T6) months of intervention.

**Results:**

The MFG and COIG were effective at reducing pain after six months (MFG: 2.5–4.5 CI, *p* = 0.001; COIG: 1.5–3.5 CI, *p *= 0.011). The FFI and FHSQ-Br showed improvements with MFG and COIG after T6 (MFG: 13.7–15.4 CI, *p* = 0.010; COIG: 11.3–15.0 CI, *p* = 0.001). The 6MWT increased with MFG (589.3–622.7 CI) and COIG (401.3–644.7 CI) and foot pronation was decreased after T3 and T6 MFG (FPI Right: 4.2–5.4 CI; Left: 3.6–5.4 CI) COIG (FPI Right: 3.4–6.8 CI; Left: 3.3–5.7 CI). The contact area reduced on forefoot and rearfoot with MFG and GOIG and midfoot and rearfoot with MFG. Maximum force was reduced on foot with MFG after T3 and T6. The peak pressure was reduced on the forefoot with MFG and COIG and on midfoot and rearfoot with MFG.

**Conclusions:**

The mechanical treatment with customized insole and minimalist flexible footwear during gait training program during six months in women with calcaneal spur reduced the calcaneus pain, increased function and health feet and reduced plantar load on the rearfoot, midfoot and forefoot. However, the footwear alone was more effective than when combined customized insole, given the greater efficacy on clinical and biomechanical aspects.

**Trial registration:**

ClinicalTrials.gov NCT03040557 (date of first registration: 02/02/2017).

## Introduction

The plantar calcaneal spurs (PCS) are typically described as bony outgrowths arising just anterior to the medial calcaneal tuberosity [1], defined as bone tissue projections of larger than 1 or 2 mm by microscopy or X-ray image assessment [1–3]. The prevalence of PCS in young to middleaged population is of 20–60% with age between 40 and 55 years [4, 5], with positive correlation for women in average 30 years [6, 7], but this rate increase with older age to 55–78% [6]. Studies have proposed that this age-dependent difference may be due to the foot biomechanics changes [7–9].

Calcaneal pain and foot functional impairment, specially during walking, are the aspects that lead the patients with this disease to seek some type of conservative treatment [10, 11]. The pathophysiology and etiology of PCS is poorly understood and several theories have been proposed [1, 12]. Traditional explanation, suggests that repetitive traction, due tension force on plantar fascia leads to inflammation and reactive ossification with formation of bone spurs [13–16]. Evidence to support this hypothesis can be derived from studies, which have shown that plantar fascia tension increases with reduction longitudinal arch, i.e., pronation feet in patients with PCS [11, 12, 17, 18] and that the heel pain are more likely to be flatfooted [19–21]. However, the accuracy of this hypothesis has also been varied and questioned by studies, which have shown that: (i) Patients with PCS may have an adaptive response of the repetitive vertical forces [2, 12, 22], in which promote the trabecular pattern predominantly perpendicular to the long axis of the spur and the weightbearing surface [2, 22]; (ii) Another evidence have revealed association of PCS with age, increase body mass index (BMI), decreased dorsiflexion at the ankle and prolonged periods of standing [21, 23]; (iii) Most of the studies in patients with PCS has hypothesized to relationship with the decrease the elasticity or atrophy of the heel fat pad for disease and heel pain [19, 20, 24–26] and (iv) Current hypothesis with fundamental clinical rationale has been directed to inadequate shoes, ie, with reduction feet mobility or excessive foot pronation, due presence of the dense and rigid shoe, are extrinsic biomechanical factors for cause and progression of the CS [17, 27, 28].

Some studies that focused on the rationale of the first hypothesis have shown the therapeutic effect of the insole to reduce pain and improve plantar pressure on the rearfoot in patients with plantar fasciitis [29–31]. Comparative, controlled, non-blind cross-sectional study evaluating three types of corrective insoles: orthotics, orthotics, bone spur pads and flat insoles, in patients with chronic plantar fasciitis, showed that pre-fabricated orthotics and customized orthotics reduced rearfoot peak forces on both sides while bone spurs heel pad increase rearfoot peak pressures [29]. Prefabricated and customized orthotics are useful in distributing pressure uniformly over the rearfoot region. Another retrospective pilot study with 10 patients with plantar fasciitis, duration 5 weeks only, evaluated one type of insoles: heel pad (medial wedge with a customized insertion of low-density) for the reduction of heel pain. According to the authors after 5 weeks of heel pad and orthotic use, all patients showed a reduction in pain, with the overall reduction being highly significant, proving to be an effective first-line treatment for the heel pain and loss of function associated with plantar fasciitis [30]. Evidence from a single-group study with comparative effect (pre and post-intervention) and duration between 12 to 17 days, showed that custom semirigid foot orthotics may significantly reduce pain experienced during walking and may reduce more global measures of pain and disability for patients with chronic plantar fasciitis [31]. Despite the promising efficacy of the insole in patients with plantar fasciitis, there are no studies in the literature with clinical trials, in the short (3 months) and long term (six months), in patients with calcaneal spurs. This clinical understanding is necessary to verify, in fact, the therapeutic effect on the pain and function of the feet of these patients affected by the disease, as well as the vertical force mechanics by the distribution of the plantar load, with the use of customized insole or flexible footwear and without high heels (minimalist) for a better strategy of mechanical treatment of the disease.

Only two recent studies, in 2021, were found in the literature with the clinical trial design, in the short term (two and a half months – 12 weeks) using the insole combined with the exercise booklet [32] and in the long term ( six months) with the use of the insole, combined shock therapy [33] for patients with plantar fasciitis, whose results showed the effectiveness of the insole to reduce pain and increase function of the feet [32, 33]. However, to date, no clinical studies have been observed in patients with calcaneal spurs in the use of insole combined with flexible shoes without high heels, a fact that shows the clinical relevance of the present study.

With the rational about footwear and based on the third and fourth hypothesis, as well as the traditional explanation, more recently, promising and effective results, in the short and long term, have showed the use of an inexpensive, flexible, non-heeled footwear (minimalist) to reduced significantly vertical forces and pain relief during gait and daily activities to descending stairs in women with inflammatory and degenerative diseases [29–38]. The rationale for this footwear is explained by capable of simulating barefoot gait and improved feet flexibility resulting in an adequate dissipation of the vertical forces on the plantar surface [37, 38]. Contrary to this rationale, clinical trials targeting traditional footwear, high-heeled and rigid soled or combined with custom-made foot insoles, in patients with PCS were performed [39, 40]. They have showed therapeutic effect to decrease pain and reduce plantar force in the forefoot [39] and rearfoot [40]. However, review study revealed that high-heeled footwear can promote some negative effects, such as increase muscle activity of the ankle plantar flexors and a possible shunting effect of increased plantar pressures to other regions of the foot when reducing pressure at the forefoot [41]. Clinical trials have show that high-heeled footwear increase plantar load on the first metatarsal [42] and reducing ankle mobility [41].

Observing previous knowledge about the negative effects of traditional footwear (high-heeled and rigid soled) and the verifying the promising effects of inexpensive, minimalist flexible footwear to reduced vertical forces on the plantar surface, pain relief and improved feet flexibility during gait of the women with inflammatory and degenerative diseases, our hypothesis was that use minimalist flexible footwear combined customized orthopaedic insole in women with PCS could promote: relieve calcaneus pain; improve feet function in daily living activities and reduce plantar load during gait. This understanding can help doctors in the indication of an effective mechanical treatment with the use of shoes and insoles to relieve pain and increase the function of the feet of patients affected by the disease, as well as its main causal factor for the better distribution of impact forces received during gait. In addition, patients would be better treated, especially in public health sectors, due to the low cost of footwear or personalized insole. Our aim is to investigate the effectiveness of mechanical treatment with customized insole and minimalist flexible footwear during gait training program in women with calcaneal spur.

## Patients and methods

### Study design and interventions groups

This was a randomised clinical trial with concealed allocation, blinding of assessors and intention-to-treat analysis with a 6-month intervention follow-up period in women diagnosed with PCS. Study setting was the biomechanics laboratory. All patients with PCS allocated to the intervention groups receive the minimalist flexible footwear (MFG) or customized othopedic insole combined minimlist flexible footwear (COIG) on the first day and have to use it for three and six months consective to gait training program, for at least six hours daily, seven days a week (42 h weekly). The patients allocated to the control group (CG) do not receive interventions, only standard protocol with footwear guidelines for walking. All patients are assessed at baseline condition (T0) and after three (T3) and six months (T6-end of intervention).

### Participants centre and selection criteria

This study started in January of the 2017 and finished in December 2019. The participants were recruited from three settings: (a) community health center; (b) orthopaedic ambulatory in the University Hospital. All potential patients were interviewed by telephone and, when selected, were assessed by a orthopedist who was blind to the patient’s allocation.

**Fig. 1:**
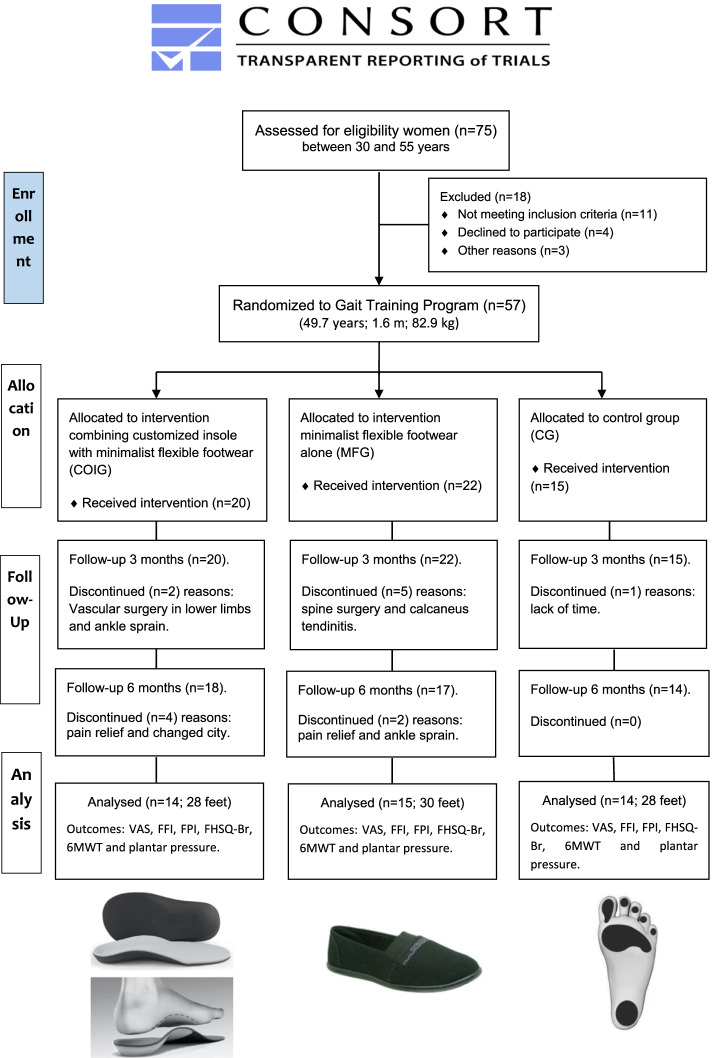
Flow Diagram of participants on trial clinical and outcomes analyzed

The participants diagnosed with PCS (ICD-10-CM code for calcaneal spur) were confirmed on physical examination (pain and plantar fascia stretch) performed by the accompanying orthopaedist doctor and also by X-ray and ultrasonography (heel spurs confirmation—between 2 and 8.0 mm). All patients with PCS were type B spurs stretched forward from the plantar fascia insertion to extend distally within the plantar fascia [10]. Forty-three women were recruited and allocated in interventions groups: twenty-nine with PCS allocated to MFG (*n* = 15) and COIG (*n* = 14) and fourteen to control CG (*n* = 14) (Fig. 1). It is important to emphasize that in this study only women were evaluated, because PCS occurs significantly more frequently in females with 34% while men are around 31% [43].

The eligibility criteria were: women aged between 30 and 55 years, body mass index (BMI) less than 35 kg/m^2^, without history of surgical (knee, ankle and hip) or muscle injury in the last 6 months and without any diagnosed neurological and rheumatological disease [10, 11, 37], difference in leg length discrepancy greater than 1 cm, rigid hallux [11] and indication for fasciotomy [11, 37]. Walk independently for at least 6 h a day without orthoses to carry out their daily activities [36, 37], withou arthroplasty and/or lower limb orthoses or indication of arthroplasty during the intervention period and not have received corticosteroid injection in the heel in the previous periods (three and six months). In addition patients could not present: ankle joint instability, dementia or inability to give consistent information and using the minimalist flexible footwear or similar shoes for more than 25 h per week. During the study, concomitant treatments, such as physical therapy, shock waves and/or acupuncture were not allowed to avoid bias in interpreting the therapeutic effects of footwear and insole as intervention. The use of paracetamol 500 mg every four hours was permitted for intervention groups for the control of pain, according to medical indication [36–38].

All participants provided written consent, based upon ethical approval by the Human Research Board of the University Local (number: 1.074.141). The ethical safety principles were: voluntary participation, informed consent, anonymity, data confidentiality, absence of potential harm to health, and results communication. Data access and storage are kept with National Health and Medical Research Council guidelines, as approved. This trial is registered in Clinical Trials (number NCT 03040557, date of first registration: 02/02/2017).

### Randomization and blinding

The randomization schedule was prepared using Clinstat software by an independent researcher who was not aware of the numeric code for the control and intervention groups [36]. A numeric block randomization sequence is kept in opaque envelopes. After the patients’ agreement and assignment to participate in the research, the allocation into the groups was made by another independent researcher, who was also unaware of the codes. Only the physiotherapist responsible for the clinical trial knows who is receiving the intervention. The initial and final clinical examinations were carried out by a orthopedist who was blind to the patient’s allocation. One physical therapist was responsible for all clinical, functional, and biomechanical assessments. Another physical therapist was responsible for monitoring the use of the interventions by telephone. Both physiotherapists were blind to treatment allocation until that analysis has been completed [36, 37].

### Outcome measures

*The primary outcome were*: foot pain by Visual Analoge Scale (VAS) and foot functionality throught questionnaires: Foot Function Index-FFI, Foot Health Status Questionnaire-FSHQ-Br and 6-min walk test-6MWT. The FFI evaluates the feet functional disability. It contains 23 questions subdivided into three domains: foot pain (9 items), difficulty (9 items) and functional limitation (5 items). The final score varies from 0 to 100, the higher the score, the worse is the condition, with the reliability and validity [44]. The FHSQ-Br evaluates foot health. This instrument contains 29 questions divided into three sessions. Session I evaluates foot health (foot pain, function, footwear and general foot health). Session II assesses general health status (physical activity, social capacity and vigor). Session III contains general patient demographics. The score varies from 0 to 100, being worse and better, respectively, with the reliability and validity [45]. The 6-min walk test (6MWT) for the assessment of functional capacity. The women walked at maximum speed for 6 min along a 22-m track, and the total distance and turn were recorded, with the reliability and validity [46].

*The Secondary outcomes was*: the plantar pressure distributions during gait using pressure platform system (Loran® Sensor Medica Inc., Italy), with four sensors inside and with dimensions of 3240 mm in length, 620 mm in width, 20 mm in height, 29 kg in weight, incorporating capacitance transducer sensors (4 sensors/cm2) at a frequency of 100 Hz. Previously, the acquisition of plantar pressure, the feet posture was verified statically using a clinical tool FPI-6 to quantify the degree to which the foot can be considered supinated (-1 to − 5), pronated (+ 6 to + 10), or normal (0 to + 5). Each foot was assessed and ranked as supinated, normal, or pronated by the sum of the FPI-6 criteria [47].

For the acquisition of gait was performed on patients' habituation to walking barefoot and to the data collection environment. The patient walked freely through a 20-m walkway, with the pressure platform fixed in the center of this walkway. It was oriented to the patient to walk in a natural way (performed in their daily lives) [37, 38]. The cadence was monitored by a metronome, but not controlled, ranging from 100 to 125 steps per minute [37]. After habituation, 3 attempts were obtained. Approximately 12 steps were acquired and the mean value per participant was used for statistical. The contact area (cm2), maximum force (N) and peak pressure (kPa) over the four plantar areas of the foot: medial and lateral rearfoot (30% of the foot length), midfoot (30% of the foot length), and forefoot and toes (40% of the foot length) were recorded.

### Intervention Protocol

**Fig. 2:**
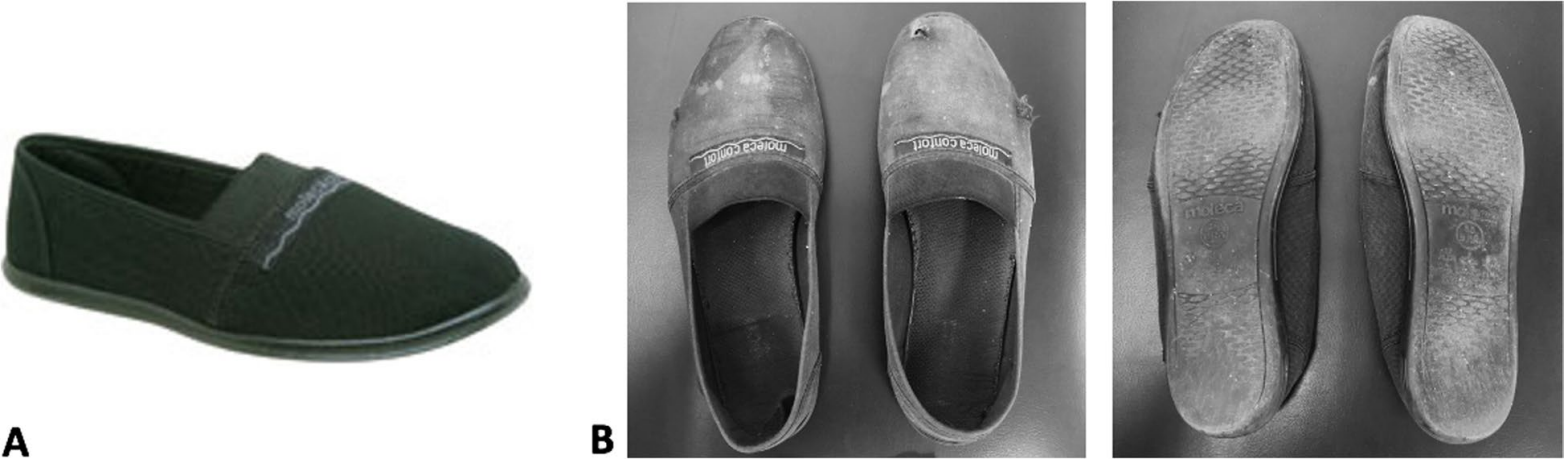
**A** Baseline of the flexible, minimalist and low-cost footwear; **B** Flexible, minimalist and low-cost footwear wear after six months of intervention

The intervention was based on gait training program with daily use of the intervention minimalist flexible footwear-MFG (Moleca®-for three and six months, for at least 42 h per week (six hours a day, seven days a week) [37, 38] (Fig. 2). This minimalist flexible footwear (Calçados Beira Rio S.A., Novo Hamburgo, RS, Brazil) was a low-cost women double canvas, flexible, flat, walking shoe without heel drop, with a 5-mm anti-slip rubber sole and a 3-mm internal wedge of ethylene vinyl acetate. Its mean weight is 0.172 ± 0.019 kg, ranging from 0.091 to 0.182 kg depending on the shoe size [32]. If there are tears or holes in the shoes, they are replaced with a new pair [37, 38].

**Fig. 3:**
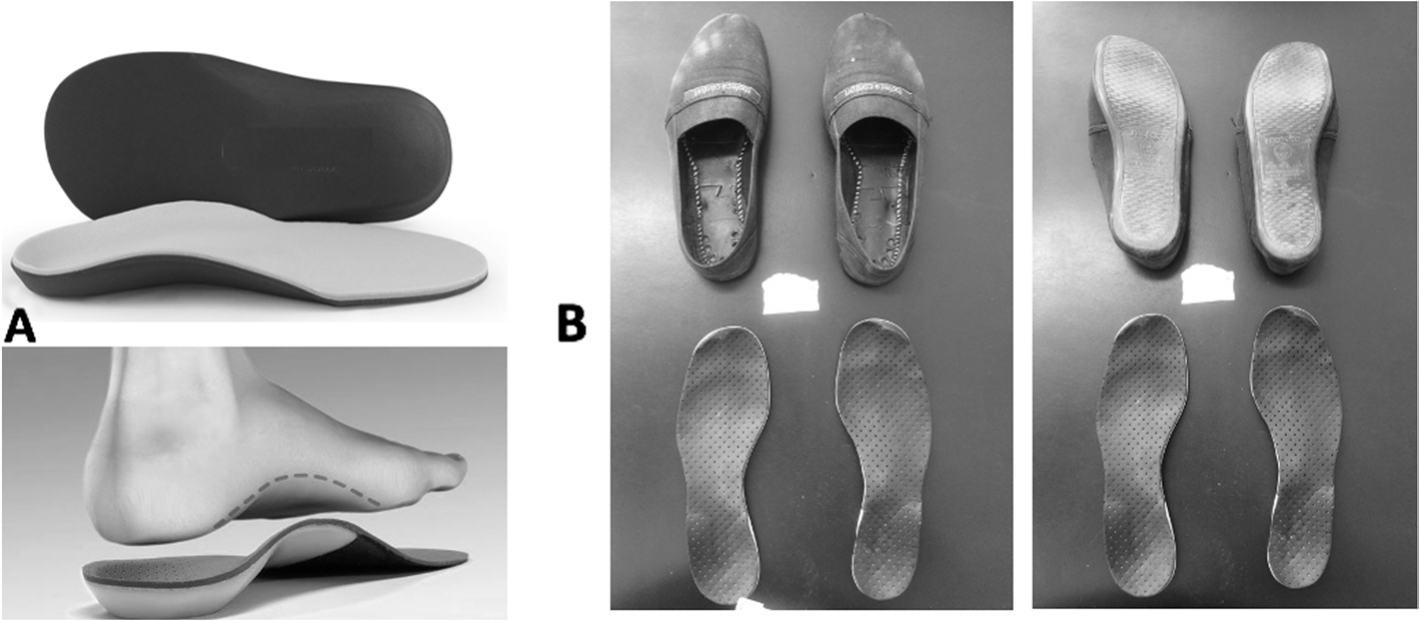
**A** Baseline of the customized orthopaedic insole, all contact with wedge at the side edge of the heel; **B** Customized orthopaedic insole, all contact with wedge at the side edge of the heel inserted in flexible, minimalist footwear, and wear after six months of intervention

The patients with PCS allocated in the intervention customized orthopaedic insoles combined minimalist flexible footwear-COIG, also used daily. The customized insoles with total contact were made for each patient individually with a wedge for the heel region. The insole was made with the patient standing on a foot foam mold, from which the EVA insole was made (Fig. 3). The intervention through during gait training program was for three and six months, for at least 42 h per week (six hours a day, seven days a week). During the intervention period, patients from the CG should not wear minimalist flexible footwear or similar minimalist shoes and customized insole, but they continue to receive their recommended health care and painkiller medication at the hospital. At the end of the intervention period, all CG patients also receive a free pair of minimalist flexible footwear with or without customized insole [37].

Every two weeks, the same physiotherapist makes phone calls to the intervention groups: MFG or COIG patients in order to verify adherence to the treatment and the correct filling out of the diary. After three and six months of intervention, the patients have their pair of footwear and insoles photographed and checked for natural wear of the shoe with its daily use (Fig. 3).

### Sample size and statistical analysis

The sample size calculation of the 46 women, based upon the foot pain variable by the effect size (difference between 2 groups: mean of treatment group minus the mean of the control group and dividing it by the standard deviation of one of the groups), was carried out using G-Power 3.0 software, considering a moderate effect size (*F* = 0.30), a statistical power of 80%, and a significance level of 5% [48, 49].

The statistical analysis were be based on intention-to-treat analysis, and two-way ANOVAs for repeated measures (baseline-T0, and after three-T3 and six months-T6) and between groups: MFG, COIG and CG (α = 5%), followed by Tukey’s post-hoc tests. Effect size with Cohen’s D between T0 and after six (T6) months of intervention, for which the values of 0.20–0.39, 0.40–0.79 and > 0.80 were considered to be small, medium, and large effect sizes, respectively [48, 49].

## Results

A total of 57 women were interested in participation of the study, but only forty-three completed the study after six months of intervention (Fig. 1). The intervention adherence rate was 85.9%, and no exercise-related adverse effect was observed or reported. Number of drops out during the study are presented in Fig. 1, according to three and six months of intervention. The women with PCS showed the length of the heel spur ranged between 2 and 7.8 mm. The characteristics of the groups were similar and also between baseline (T0) and after three (T3) and six months (T6) of intervention (Table 1).

**Table 1:** Mean, standard deviation and comparison between women with plantar calcaneus spur (PCS) and control (CG), for anthropometric data of the baseline condition (T0) and after three (T3) and six months of intervention (T6) with minimalist flexible footwear (MFG) and customized orthopaedic insole combined minimalist flexible footwear (COIG)

**Variable**	**Intervention** **(months)**	**PCS—MFG** **(*n* = 15)**	**PCS—COIG** **(*n* = 14)**	**Control—CG** **(*n* = 10)**	p*
**Age (years)**	T0	48.9 ± 9.4	50.3 ± 5.8	47.8 ± 8.6	0.730
**Height (m)**	T0	1.61 ± 0.1	1.62 ± 0.1	1.63 ± 0.1	0.394
**Body mass (kg)**	T0	82.1 ± 14.1	83.6 ± 13.9	73.2 ± 12.6	0.160
	T3	80.4 ± 12.8	81.0 ± 12.6	73.0 ± 12.4	0.280
	T6	78.4 ± 11.6	80.8 ± 12.5	73.4 ± 12.5	0.321
	p	0.151	0.068	0.885	
**Body mass index (kg/m2)**	T0	32.1 ± 7.0	32.2 ± 4.3	27.5 ± 4.5	0.082
	T3	31.4 ± 4.7	31.2 ± 4.2	27.8 ± 4.1	0.068
	T6	30.5 ± 4.5	31.1 ± 4.2	28.6 ± 4.6	0.064
	p	0.769	0.073	0.695	

^*^ Analysis of variance two-way (inter-groups) and repeated measures, considering significant differences *p* < 0.005

### Primary outcomes

Both interventions MFG and COIG with large effect size promoted improvements in foot pain and functionality in women with PCS. Inter-groups, the MFG was better at improving feet function to when compared COIG and CG after T6. Another important finding was the decreasing FPI after T3 and T6 with MFG and COIG, with large effect size to MFG and low to COIG (Table 2).

**Table 2:** Mean, standard deviation, effect size and inter-groups comparisons: minimalist flexible footwear (MFG) and custom orthopaedic insole combined minimalist flexible footwear (COIG) and intra-moments of the intervention: T0 (baseline), T3 and T6 (months) for primary outcomes: pain (EVA), FFI (Foot Function Index) and FPI-6 (Foot Posture Index) in women with plantar calcaneus spur (PCS)

**Variable**	**Intervention** **(Groups)**	**Baseline-T0 (1)**	**Three months-T3** **(2)**	**Six months-T6** **(3)**	***Effect size***	***95%*** ***CI***	p*
**Foot pain (cm)**	MFG (1)	9.5 ± 0.8	5.2 ± 1.8	3.5 ± 1.6	0.87	8.6–10^1^ 4.1–6.3^2^ 2.5–4.5^3^	0.001^1–2; 1–3^
	COIG (2)	9.3 ± 1.0	3.4 ± 2.1	2.5 ± 1.7	0.88	8.7–9.8^1^ 2.2–4.4^2^ 1.5–3.5^3^	0.011^1–2; 1–3^
	p^#^	0.468	0.07	0.118			
**Comfort footwear (cm)**	MFG (1)	7.8 ± 2.1	7.8 ± 1.7	8.2 ± 1.7	0.20	6.5–9.1^1^ 5.9–9.8^2^ 6.6–10^3^	0.856
	COIG (2)	9.0 ± 0.8	8.8 ± 1.5	9.1 ± 1.0	0.11	8.5–9.5^1^ 8.3–10^2^ 9.4–10^3^	0.790
	p^#^	0.015	0.038	0.031			
**Foot Function Index – (FFI)** **(0—100)**	MFG (1)	82.0 ± 14.6	15.7 ± 1.3	15.0 ± 1.4	0.45	72.2–91.3^1^ 14.3–15.8^2^ 13.7–15.4^3^	0.010^1–2; 1–3^
	COIG (2)	78.8 ± 11.4	14.1 ± 2.5	13.2 ± 3.3	0.36	72.3–85.4^1^ 12.7–15.6^2^ 11.3–15.0^3^	0.001^1–2; 1–3^
	CG (3)	24.2 ± 5.1	25.6 ± 6.8	26.3 ± 8.1	0.30	20.2–29.6^1^ 23.5–31.5^2^ 19.3–33.2^3^	0.773
	p*	0.010^1–3;2–3^	0.759	0.005^1–3;2–3^			
**Foot Posture Index (FPI-6) Right (total score)**	MFG (1)	6.0 ± 2.6	4.9 ± 1.8	4.8 ± 1.0	0.60	4.3–7.6^1^ 3.8–6.0^2^ 4.2–5.4^3^	0.047^1–3^
	COIG (2)	6.0 ± 4.1	5.8 ± 2.2	5.1 ± 2.9	0.25	3.8–8.4^1^ 4.6–7.1^2^ 3.4–6.8^3^	0.034^1–3^
	CG (3)	3.7 ± 2.2	3.9 ± 2.5	4.0 ± 2.7	0.12	3.6–6.5^1^ 3.9–5.7^2^ 3.4–5.0^3^	0.988
	p*	0.024^1–3;2–3^	0.106	0.340			
**Foot Posture Index (FPI-6) Left (total score)**	MFG (1)	6.2 ± 4.9	5.7 ± 4.6	5.5 ± 2.1	0.27	4.4–7.7^1^ 2.9–5.5^2^ 3.6–5.4^3^	0.045^1–3^
	COIG (2)	6.0 ± 5.1	5.0 ± 4.8	5.0 ± 2.5	0.25	4.6–8.7^1^ 4.3–6.8^2^ 3.3–5.7^3^	0.039^1–3^
	CG (3)	4.7 ± 3.6	4.3 ± 3.0	4.8 ± 2.9	0.04	4.6–8.5^1^ 3.6–6.4^2^ 4.1–6.0^3^	0.963
	p*	0.013^1–3;2–3^	0.181	0.564			

^*^Analysis of variance, repeated measures, intra-assessments: baseline T0 (1) and after T3 (2) and T6 (3) months of intervention and inter-groups (MFG, COIG and CG), followed by Tukey's post hoc, significant differences *p* < 0.005 and 95% CI. Effect size with Cohen’s D between T0 and after six (T6) months of intervention. ^#^Analysis by t Student between gropus (MFG and COIG) to T0, T3 and T6

Regarding the FSHG-Br significant improvements can be observed after T3 and T6 with COIG and MFG. Inter-groups, only MFG showed improvement after T6 compared to CG. The pain showed improvements after T3 and T6 with COIG and MFG. Inter-groups, the MFG and COIG showed effective improvements after T6 compared to CG. The foot fucntion and general foot health showed significant improvements after T3 and T6 with MFG and COIG. Inter-groups, the MFG and COIG increased the health and function of the feet after T3 and T6. To the footwear, MFG and COIG, showed effectiveness after T3 and T6, with a large effect size for MFG and moderate for COIG. Inter-groups, the MFG and COIG showed reduced compared to the CG after T3 and T6 (Table 3). The six-minute walk test improved with COIG and MFG increased after T3 and T6, with moderate effect size (Table 4).

**Table 3:** Mean, standard deviation, effect size and inter-groups comparisons: minimalist flexible footwear (MFG) and custom orthopaedic insole combined minimalist flexible footwear (COIG) and intra-moments of the intervention: T0 (baseline), T3 and T6 (months) for primary outcomes: FSHQ-Br (Foot Health Status) in women with plantar calcaneus spur (PCS)

**Variable**	**Intervention** **(Groups)**	**Baseline-T0 (1)**	**Three months-T3** **(2)**	**Six months-T6** **(3)**	***Effect size***	***95%*** ***CI***	p*
**Foot Health Status – (FSHQ-Br) (0—100)**	MFG (1)	48.8 ± 11.0	59.6 ± 14.1	90.7 ± 9.5	0.48	10.5–53.2^1^ 15.7–61.4^2^ 92.3–98.5^3^	0.001^1–3;2–3^
	COIG (2)	48.3 ± 17.0	60.1 ± 16.7	94.5 ± 6.9	0.45	29.9–62.2^1^ 35.2–70.5^2^ 89.3–95.0^3^	0.010^1–3;2–3^
	CG (3)	84.4 ± 7.9	85.1 ± 6.7	85.4 ± 6.9	0.13	80.9–92.3^1^ 85.7–89.3^2^ 82.5–91.4^3^	0.839
	p*	0.005^1–3;2–3^	0.096	0.030^1–3^			
**Foot Pain**	MFG (1)	18.5 ± 1.6	8.8 ± 2.2	5.4 ± 1.8	0.76	17.6–19.7^1^ 7.5–10.3^2^ 4.4–7.0^3^	0.011^1–2; 1–3;2–3^
	COIG (2)	17.6 ± 1.4	8.3 ± 3.5	7.0 ± 4.9	0.72	16.7–18.9^1^ 6.2–10.4^2^ 4.1–9.9^3^	0.001^1–2; 1–3^
	CG (3)	17.8 ± 2.5	17.6 ± 2.5	18.0 ± 2.8	0.07	16.8–18.0^1^ 15.5–18.9^2^ 14.2–18.5^3^	0.969
	p*	0.015^1–3;2–3^	0.019^1–3;2–3^	0.021^1–3; 2–3^			
**Foot Function**	MFG (1)	1.7 ± 1.0	3.7 ± 0.8	17.6 ± 2.9	0.73	0.4–2.9^1^ 1.0–7.4^2^ 15.6–19.3^3^	0.001^1–2; 1–3^
	COIG (2)	2.7 ± 1.6	4.0 ± 3.5	16.3 ± 4.8	0.38	1.4–3.9^1^ 1.8–6.0^2^ 13.5–19.4^3^	0.001^1–2; 1–3^
	CG (3)	24.8 ± 0.7	25.0 ± 0.9	25.0 ± 0.9	0.24	19.8–27.5^1^ 21.8–29.2^2^ 23.5–30.2^3^	0.661
	p*	0.010^1–3;2–3^	0.001^1–3;2–3^	0.040^1–3;2–3^			
**Footwear**	MFG (1)	5.5 ± 2.6	5.0 ± 3.0	3.2 ± 1.4	0.90	4.0–7.2^1^ 0.2–5.3^2^ 0.3–3.8^3^	0.015^1–3^
	COIG (2)	5.9 ± 2.5	4.5 ± 3.3	4.2 ± 3.6	0.54	4.4–7.5^1^ 1.2–6.0^2^ 0.4–7.4^3^	0.027 ^1–3^
	CG (3)	9.6 ± 3.7	10.0 ± 4.1	10.5 ± 4.7	0.21	6.6–10^1^ 6.1–9.8^2^ 7.5–10.8^3^	0.890
	p*	0.020^1–3;2–3^	0.016^1–3;2–3^	0.001^1–3;2–3^			
**General foot health**	MFG (1)	3.6 ± 1.4	2.6 ± 1.8	9.3 ± 1.5	0.40	1.6–3.8^1^ 1.5–4.7^2^ 8.3–10.2^3^	0.001^1–2; 1–3^
	COIG (2)	2.6 ± 1.5	3.2 ± 1.9	9.6 ± 0.8	0.58	1.8–3.4^1^ 1.5–5.2^2^ 8.2–10.0^3^	0.007^1–2; 1–3^
	CG (3)	13.9 ± 3.8	13.9 ± 3.9	14.0 ± 4.1	0.02	10.8–14.5^1^ 10.5–14.7^2^ 10.8–15.0^3^	0.740
	p*	0.001^1–3;2–3^	0.001^1–3;2–3^	0.014^1–3;2–3^			

^*^Analysis of variance, repeated measures, intra-assessments: baseline T0 (1) and after T3 (2) and T6 (3) months of intervention and inter-groups (MFG, COIG and CG), followed by Tukey's post hoc, significant differences *p* < 0.005 and 95% CI. Effect size with Cohen’s D between T0 and after six (T6) months of intervention

**Table 4:** Mean, standard deviation, effect size and inter-groups comparisons: minimalist flexible footwear (MFG) and custom orthopaedic insole combined minimalist flexible footwear (COIG) and intra-moments of the intervention: T0 (baseline), T3 and T6 (months) for outcomes: six-minute walk test (6MTW) in women with plantar calcaneus spur (PCS)

**6MWT**	**Intervention** **(Groups)**	**Baseline -T0 (1)**	**Three months -T3** **(2)**	**Six months-T6** **(3)**	***Effect size***	**95%** **CI**	p*
**Travelled distance (m)**	MFG (1)	283.8 ± 84.5	534.0 ± 99.6	606.0 ± 33.4	0.51	232.8–334.9^1^ 401.3–666.7^2^ 589.3–622.7^3^	0.001^1–2; 1–3^
	COIG (2)	272.0 ± 79.7	488.2 ± 91.2	523.0 ± 37.2	0.40	227.8–316.2^1^ 386.6–589.8^2^ 401.3–644.7^3^	0.001^T1−2; 1–3^
	CG (3)	741.2 ± 56.0	723.2 ± 52.9	742.8 ± 44.7	0.04	532.8–764.0^1^ 601.3–780.5^2^ 689.5–762.5^3^	0.764
	p*	< 0.001^1–3;2–3^	0.001^1–3;2–3^	0.037^1–3;2–3^			
**Number of turns (n)**	MFG (1)	9.5 ± 2.8	17.8 ± 3.6	20.2 ± 1.4	0.48	7.7–11.1^1^ 13.3–22.2^2^ 19.6–21,7^3^	0.001^1–2; 1–3^
	COIG (2)	9.1 ± 2.7	17.3 ± 3.5	19.4 ± 3.9	0.30	7.6–10.5^1^ 15.2–20.1^2^ 16.3–21.8^3^	0.001^T1−2; 1–3^
	CG (3)	24.1 ± 1.6	23.8 ± 1.5	24.2 ± 1.4	0.06	20.6–28.6^1^ 19.9–27.2^2^ 20.6–27.7^3^	0.568
	p*	< 0.001^1–3;2–3^	0.004^1–3;2–3^	0.006^1–3;2–3^			

^*^Analysis of variance, repeated measures, intra-assessments: baseline T0 (1) and after T3 (2) and T6 (3) months of intervention and inter-groups (MFG, COIG and CG), followed by Tukey's post hoc, significant differences *p* < 0.005 and 95% CI. Effect size with Cohen’s D between T0 and after six (T6) months of intervention

### Secondary outcome

Plantar pressure distribution revealed that: A) Contact area: forefoot showed decreasing after T6 with COIG and MFG. In the midfoot, only MFG showed decreases after T3 and T6. Inter-groups, MFG showed decreases compared COIG. Lateral rearfoot, only the MFG decreased after T6. B) Maximum force: forefoot showed effective decrease with MFG after T6. Midfoot showed effective decrease with both MFG and COIG after T6. Inter-groups, MFG showed decrease after T6 when compared CG and GOIC. Inter-group, after T6, MFG showed decrease compared CG; C) Peak pressure: forefoot showed decreases after T3 and T6 with MFG and COIG. Inter-groups, MFG and COIG promoted decrease compared to CG after T6. Midfoot showed decreases with use MFG after T6. Inter-groups, mifoot showed decrease with MFG and increase to GOIC when compared to CG. Medial and lateral rearfoot, both MFG and COIG showed effective decrease after T6. Inter-groups, medial and lateral rearfoot with MFG were effective to decrease in relation COIG (Table 5).

**Table 5:** Mean, standard deviation, effect size and comparasions intra-moments of the intervention: T0, T3 and T6 and inter-groups: minimalist flexible footwear (MFG), custom orthopaedic insole combined minimalist flexible footwear (COIG) and control (CG) for plantar pressure distribution of the women with calcaneus spur

**Variable**	**Intervention**	**Baseline -T0 (1)**	**Three months -T3** **(2)**	**Six months -T6** **(3)**	***Effect size***	***95% CI*** ***T6***	p*
**Forefoot** **Contact area (cm)**	MFG (1) COIG (2)	12.3 ± 1.4 16.4 ± 9.1	11.4 ± 1.4 12.2 ± 1.5	11.1 ± 1.4 11.4 ± 1.2	0.85 0.77	10.1–12.1 10.8–12.0	0.024^1–3^ 0.012^1–3^
	CG (3)	11.5 ± 1.7	11.6 ± 1.5	12.1 ± 1.3	0.39	10.5–12.7	0.849
	p*	0.022^1–2;1–3;2–3^	0.614	0.392			
**Midfoot** **Contact area (cm)**	MFG (1) COIG (2)	29.5 ± 12.9 34.4 ± 15.4	15.2 ± 11.1 31.5 ± 14.4	14.9 ± 11.3 26.9 ± 10.0	0.92 0.57	11.8–22.8 19.8–31.0	0.010^1–2;1–3^ 0.208
	CG (3)	23.6 ± 11.9	23.6 ± 11.8	22.9 ± 12.2	0.05	15.4–32.1	0.988
	p*	0.001^1–3;2–3^	0.041^1–2;2–3^	0.008^1–2^			
**Medial Rearfoot** **Contact area (cm)**	MFG (1) COIG (2)	20.8 ± 1.8 21.4 ± 2.9	20.5 ± 2.1 21.5 ± 3.3	20.0 ± 2.6 20.3 ± 2.4	0.35 0.41	18.0–21.8 19.0–21.4	0.548 0.352
	CG (3)	20.4 ± 3.6	19.9 ± 4.3	20.1 ± 3.0	0.10	17.8–22.9	0.961
	p*	0.001^1–3;2–3^	0.623	0.965			
**Lateral Rearfoot** **Contact area (cm)**	MFG (1) COIG (2)	21.7 ± 1.8 21.8 ± 2.8	20.7 ± 1.7 23.0 ± 4.2	19.8 ± 1.9 21.3 ± 2.2	0.91 0.19	18.4–21.1 20.0–22.4	0.026^1–3^ 0.227
	CG (3)	20.6 ± 3.9	21.2 ± 3.3	21.8 ± 3.8	0.31	17.7–23.4	0.972
	p*	0.001^1–3;2–3^	0.094	0.171			
**Forefoot** **Maximum force (N/BW)**	MFG (1) COIG (2)	0.19 ± 0.02 0.19 ± 0.04	0.20 ± 0.03 0.18 ± 0.02	0.18 ± 0.04 0.18 ± 0.03	0.31 0.28	0.16–0.19 0.15–0.19	0.032^1–3^ 0.575
	CG (3)	0.18 ± 0.03	0.19 ± 0.03	0.19 ± 0.02	0.33	0.16–0.21	0.985
	p*	0.401	0.343	0.852			
**Midfoot** **Maximum force (N/BW)**	MFG (1) COIG (2)	0.26 ± 0.14 0.32 ± 0.21	0.12 ± 0.11 0.31 ± 0.23	0.11 ± 0.10 0.23 ± 0.13	0.91 0.51	0.11–0.18 0.16–0.28	0.023^1–3^ 0.029^1–3^
	CG (3)	0.18 ± 0.03	0.19 ± 0.03	0.19 ± 0.04	0.28	0.10–0.22	0.904
	p*	0.001^1–2;1–3;2–3^	0.034^1–2;2–3^	0.012^1–2;2–3^			
**Medial rearfoot** **Maximum force (N/BW)**	MFG (1) COIG (2)	0.34 ± 0.07 0.33 ± 0.06	0.38 ± 0.08 0.34 ± 0.07	0.33 ± 0.06 0.31 ± 0.06	0.15 0.33	0.26–0.35 0.30–0.37	0.570 0.236
	CG (3)	0.31 ± 0.05	0.32 ± 0.05	0.32 ± 0.04	0.22	0.26–0.35	0.798
	p*	0.136	0.147	0.318			
**Lateral rearfoot** **Maximum force (N/BW)**	MFG (1) COIG (2)	0.33 ± 0.05 0.35 ± 0.09	0.34 ± 0.06 0.38 ± 0.08	0.29 ± 0.05 0.34 ± 0.06	0.80 0.13	0.25–0.32 0.32–0.38	0.085 0.353
	CG (3)	0.31 ± 0.05	0.30 ± 0.04	0.32 ± 0.07	0.16	0.27–0.35	0.787
	p*	0.257	0.004^2–3^	0.033^1–2;1–3^			
**Forefoot** **Peak Pressure (kPa)**	MFG (1) COIG (2)	277.0 ± 36.3 280.4 ± 29.4	309.5 ± 18.2 301.5 ± 21.6	290.0 ± 23.9 310.0 ± 29.0	0.42 0.92	278.6–331.0 295.6–324.4	0.023^1–2;1–3^ 0.017^1–2;1–3^
	CG (3)	315.9 ± 47.4	317.9 ± 44.6	316.5 ± 46.5	0.02	282.0–349.8	0.774
	p*	0.023^1–3;2–3^	0.812	0.046^1–2;1–3^			
**Midfoot** **Peak Pressure (kPa)**	MFG (1) COIG (2)	178.7 ± 35.2 183.1 ± 55.1	175.3 ± 12.1 190.5 ± 54.8	145.5 ± 10.1 183.2 ± 40.2	0.91 0.57	97.0–195.7 163.2–203.2	0.003^1–3^ 0.868
	CG (3)	153.3 ± 37.7	154.2 ± 37.0	152.2 ± 38.8	0.02	126.3–180.3	0.904
	p*	0.013^1–3;2–3^	0.115	0.001^1–2;1–3^			
**Medial rearfoot** **Peak Pressure (kPa)**	MFG (1) COIG (2)	280.7 ± 31.0 285.4 ± 37.6	297.3 ± 41.7 309.3 ± 30.6	268.5 ± 29.2 305.3 ± 49.8	0.40 0.45	258.0–305.6 280.5–330.0	0.037^;1–3;2–3^ 0.008^1–3;2–3^
	CG (3)	246.8 ± 31.9	247.9 ± 34.1	249.5 ± 37.6	0.07	249.0–342.2	0.912
	p*	0.001^1–3;2–3^	0.008^1–3;2–3^	0.002^–1−2; 2–3^			
**Lateral rearfoot** **Peak Pressure (kPa)**	MFG (1) COIG (2)	264.0 ± 38.0 268.1 ± 43.8	292.4 ± 41.3 298.4 ± 35.2	271.7 ± 31.6 302.2 ± 56.2	0.73 0.67	258.0–305.1 280.5–330.0	0.001^1–3^ 0.023^1–3^
	CG (3)	266.3 ± 37.1	264.5 ± 35.4	263.3 ± 34.7	0.08	249.0–342.2	0.983
	p*	0.181	0.098	0.007^–1−2; 2–3^			

^*^Analysis of variance, repeated measures, intra-assessments: baseline T0 (1) and after T3 (2) and T6 (3) months of intervention and inter-groups (MFG, COIG and CG), followed by Tukey's post hoc, significant differences *p* < 5% and 95% CI (T6). Effect size with Cohen’s D between T0 and after six (T6) months of intervention

### Discussion

To our knowledge, this is the first randomized, clinical trial that aims at mechanical treatment for PCS comparing the efficacy between minimalist flexible footwear (MFG) and custom orthopaedic insoles combined minimalist flexible footwear (COIG) as a training program for walking in women with PCS. We propose a minimalist flexible footwear as an intervention that mimics barefoot gait and, consequently, may reduce heel plantar loading in women with PCS, created for guidance better the conservative treatment with lower cost for these patients. Based on our resulted, both interventions MFG and COIG promoted improvements in foot pain, functionality and health of the feet and decrease plantar loading in women with PCS. However, differently from what we expected, the use of MFG alone was more effective on clinical aspects (pain, function and health of the feet) and biomechanics (decrease in plantar load on heel) when compared to COIG and GC.

Two recent studies, in 2021, were found in the literature with insole combined with the exercise booklet (two months) [32] and use of the insole combined shock therapy (six months) [33] for patients with plantar fasciitis. The studies showed the effectiveness of the insole to reduce pain and increase function of the feet [32, 33]. However, to date, no clinical studies have been observed in patients with calcaneal spurs in the use of insole combined with minimalist flexible footwear (without high heels), a fact that shows the clinical relevance of the present study. Previous studies have shown the efficiency and benefits of an inexpensive minimalist flexible footwear for pain reduction in degenerative diseases, such as osteoarthritis [34–38]. Another authors reveal the importance of heel pain relief in patients with plantar fasciitis and PCS, given the typical cost per clinic visit is about $50 and the average cost of prescription non-steroidal anti-inflammatory drugs-NSAIDs, a common first-line treatment to calcaneus pain reduce, is nearly $600 per patient per year [50]. Noninvasive treatments, such as custom insole on heel and night splints cost about $500 [50–52]. In this study, the costs related to footwear were on average $60 and associated with an customized insole of around $100, showing to be a conservative strategy of lower cost and effective access to patients. In addition, footwear is the basic component for walking and the worst complaint of difficulties in access and purchase by these patients. Observing our positive results for heel pain relief, we can suggest that, perhaps, clinical visits and medication use may be reduced over time using MFG or COIG, given the consecutive improvement in pain after three and six months of the intervention.

In addition to pain relief, positive therapeutic improvements in the function and health index of feet were observed with the use of MFG and COIG. It is worth mentioning, that the therapeutic effect was more effective and increased with the use of MFG alone when compared to COIG and CG. The first line of reasoning to explain the benefits of this footwear in patients with PCS, comes from the theoretical-practical explanation of its promising functional effects for reducing vertical forces on the foot and knee in patients with chronic degenerative diseases, such as knee osteoatritis [34–36], as well as in healthy women comparing footwear with sole at different heel height: flat, low and hight [41]. The second explanation has been for the reduction of the feet pronation in static conditions and reduction of the plantar pressure on heel that allowed an increase in walking distance, with a larger effect size for footwear alone (MFG), agreeing with Kuyucu et al., (2012) [53] who emphasizes the association of calcaneal spur length and clinical and functional parameters.

Studies elucidate the importance of gait function in patients with PCS to prevent abductor digiti minimi atrophy and less elasticity of the plantar fascia [25–28], as well as social isolation due to difficulty walking and limitations to daily functional activities [17, 27]. Other evidence strongly reports a foot pronation reduction to avoid repetitive stretching of the plantar fascia and accumulation and formation of the heel spur [11, 12, 17, 18]. In agreement with these reports, in this study, a significant reduction in the feet pronation can be observed, in addition to an increase in its functionality and in the distance walked, with the use of MFG and COIG, showing great effectiveness for clinical practice. The improvement in foot function and better distribution of the plantar load over the base of the feet with the minimalist footwear can also be explained by the increase in foot muscle strength that this type of footwear promotes. According to a study carried out with athletes, the minimalist shoe walking is as effective as foot strengthening exercises in increasing foot muscle size and strength. The convenience of changing footwear rather than performing specific exercises may result in greater compliance. This line of reasoning may also explain the gain in functional improvement of the feet during gait in women with foot disease, as is the case with PCS [54].

The advantages and benefits of the clinical indication for the use MFG for patients with PCS can be considered in relation to its therapeutic effects on reducing plantar pressure on heel compared COIG and CG. Contrary to this rationale, clinical trials targeting traditional footwear, high-heeled and rigid soled or combined with custom-made foot orthoses, in patients with PCS, showed therapeutic effect to decrease pain and reduce plantar pressure on forefoot [39] and rearfoot [40], however in the short term and not associating functional and health aspects of the feet.

The applicability of using MFC alone can be justified in studies with healthy individuals aimed at long-term (two years) the use of footwer with high heel soles (5 cm), which resulted in substantial increases in plantar fascia strains and increase muscle activation during gait compared with barefoot walking, with experience discomfort and and feeling of muscle fatigue [51]. Other evidence points out that footwear with high heel soles during walking can promote: higher energy costs influencing the mechanics of the lower limbs [52], increased pressure on medial forefoot [8] and other regions of the foot [41].

Randomized Controlled Trials represent the cornerstone of Evidence-Based Medicine. Based upon the rules of Good Clinical Practice, they offer many strengths. In this clinical trial study, the strengths were: the rigorous methodology used allows avoidance of bias related to confounding factors (through a control group), selection bias (through randomization with concealed allocation), interpretation bias (through double blinding of advisors) and intervention with duration of six months for detecting small, moderate to high effects that is clinically important. In addition, another strong point was the use of low-cost footwear for mechanical treatment, helping its applicability in the public health system of patients affected by the disease.

The potential limitation of this study was not to include monitoring of the fascia thickness by ultrasound and muscle strength of the feet over the six months of follow-up with both interventions, however, our main concern, in this first moment, was to evaluate variables related to symptoms, functionality and overload of the feet of women with PF. Future studies with this monitoring of the plantar fascia and muscle strength of the feet may help health professionals to better understand the physiological response of the plantar fascia when performing these mechanical foot support interventions during walking.

## Conclusion

The mechanical treatment with customized insole and minimalist flexible footwear during gait training program during six months in women with calcaneal spur reduced the calcaneus pain, increased function and health feet and reduced plantar load on the rearfoot, midfoot and forefoot. However, the footwear alone was more effective treatment than when combined customized insole, given the greater efficacy on clinical and biomechanical aspects.

## Data Availability

The datasets used and/or analyzed during the current study are available from the corresponding author on reasonable request (apribeiro@alumni.usp.br). The data are not publicly available due to their containing information that could compromise the privacy of research participants.

## Abbreviations

PCS: Plantar calcaneal spurs
MFG: Minimalist flexible footwear
COIG: Customized insole with minimalist flexible footwear
CG: Control group; T0: baseline
T3: After three six months
T6: After six months
VAS: Visual Analogue Scale
FFI: Foot Function Index
FHSQ-Br: Foot Health Status Questionnaire
6MWT: 6-Min walk test
